# Oncology nurses’ perceptions of barriers and facilitators to CAUTI prevention: a qualitative study guided by the nursing role effectiveness model

**DOI:** 10.1186/s12912-026-04978-1

**Published:** 2026-07-25

**Authors:** Filipe Paiva-Santos, Paulo Santos-Costa, Luísa Teixeira-Santos, Celeste Bastos, João Graveto

**Affiliations:** 1 School of Medicine and Biomedical Sciences (ICBAS), University of Porto, Porto, Portugal; 2 Univ Coimbra, UICISA E, ESEUC, Coimbra, Portugal; 3 Nursing Research Innovation and Development Centre of Lisbon (CIDNUR), School of Nursing, Universidade Lisboa, Lisbon, Portugal; 4 RISE-Health: Health Research Network, Nursing School of Porto University (ESEP), Porto, Portugal

**Keywords:** Urinary catheters, Catheter-related infections, Nurses, Qualitative study, Quality improvement

## Abstract

**Background:**

Catheter-associated urinary tract infection (CAUTI) is a frequent and preventable healthcare-associated infection. Nurses play a central role in CAUTI prevention, yet barriers to consistent implementation of evidence-based practices persist. In Portugal, few studies have explored nurses’ lived experiences in this area. This study aimed to explore Portuguese oncology nurses’ perceptions of barriers and facilitators to CAUTI prevention, and to identify suggestions for improving urinary catheter-related care.

**Methods:**

An exploratory qualitative study was conducted with a purposive sample of eight oncology nurses from a Portuguese oncology hospital. Data were collected through a 90-minute focus group, in February 2023, and analyzed using inductive thematic analysis. The Nursing Role Effectiveness Model informed data interpretation and theme organization.

**Results:**

Two main domains emerged: structural variables and process-related variables. Nurses identified facilitators such as continuing education, access to catheterization kits, and a positive team culture. Barriers included inconsistent material availability, gaps in supervision of delegated tasks, variability in catheterization practices, and challenges in interprofessional communication. Patient and family engagement was rarely mentioned, suggesting that CAUTI prevention was conceptualized primarily as a nurse-driven activity. Nurses valued opportunities for reflective practice and expressed interest in standardized protocols and improved documentation systems.

**Conclusions:**

Oncology nurses demonstrated strong engagement in CAUTI prevention, yet gaps remain in supervisory practices and patient involvement. Fostering reflective practice, enhancing supervisory skills, and broadening conceptions of nurse-sensitive outcomes may support more comprehensive and sustainable CAUTI prevention strategies in clinical practice.

**Clinical trial number:**

Not applicable.

## Background

Healthcare-associated infections (HAIs) remain a significant challenge to patient safety and care experience worldwide, contributing to avoidable morbidity, mortality, and financial burden [1]. Among healthcare-associated infections, catheter-associated urinary tract infection (CAUTI) remains one of the most frequent and clinically significant conditions. Despite its associated costs and morbidity, CAUTI is widely recognized as preventable through adherence to evidence-based practices [2]. In Europe, urinary tract infections account for 19.2% of all HAIs. At any given time, approximately 1.5% of hospitalized patients have a urinary tract infection, and 61.9% of these infections are associated with urinary catheter use [3], largely due to inappropriate catheter indication and management. This results in prolonged hospital stays, increased healthcare costs [4], and higher patient morbidity and mortality [5, 6]. In Portugal, the urinary catheter utilization rate is approximately 18%, with infection rates reaching up to 14.5 per 1000 catheter-days [7, 8], underscoring the need for ongoing efforts to improve prevention.

International and national guidelines emphasize a set of core interventions to reduce CAUTI incidence, including daily assessment of catheter necessity, aseptic insertion techniques, maintenance of a closed drainage system, and strict adherence to hand hygiene protocols [9]. Nurses play a pivotal role across all stages of CAUTI prevention through their proximity to patients and families, responsibility for catheter insertion and maintenance, and ability to identify early signs of complications. Beyond technical procedures, they also shape organizational culture, interprofessional communication, supervision of delegated tasks, and patient education [10, 11].

Despite this central role, the translation of guidelines into consistent clinical practice remains challenging [12]. Research highlights persistent barriers to CAUTI prevention, such as insufficient professional training, lack of material resources, fragmented interprofessional communication, and cultural resistance to behavior change [7, 13]. Moreover, the success of preventive efforts depends not only on structural and organizational factors but also on team dynamics and the extent to which patients and their families are engaged as active partners in care [10, 11].

People with oncologic disease present a particularly high vulnerability to HAIs, including CAUTI. Chemotherapy-induced immunosuppression, neutropenia, malnutrition, impaired mucosal integrity, and the frequent need for invasive devices substantially increase the risk of colonization and infection [14, 15]. In addition, recurrent hospitalization, therapeutic intensity, and the heightened likelihood of exposure to multidrug-resistant organisms make oncology settings especially sensitive to failures in infection prevention practices [16].

CAUTI prevention involves a diverse set of interacting factors [17]. The Nursing Role Effectiveness Model [18] provides a structured framework for identifying and analysing these variables within a multifactorial network. The model provides a robust framework for understanding how nursing practice influences the occurrence of HAIs by explicitly linking structure, processes, and outcomes. Structural variables, such as staffing levels, team qualifications, and care organization, shape the conditions under which nurses work, whereas care processes, including clinical surveillance, invasive device management, and adherence to aseptic practices, represent key nursing interventions [18]. Outcomes, such as HAIs, are conceptualized as indicators sensitive to the quality of these processes [18]. By articulating the relationships between organizational conditions, professional practice, and patient outcomes, the model supports the assessment of mediating mechanisms through which structural and process-related factors influence preventive practices and infection risk. This integrative perspective makes the model particularly valuable for studies aiming to explore and interpret nurses’ contributions to infection prevention and to inform structural and process-oriented interventions that enhance patient safety [19].

In Portugal, existing research on CAUTI has primarily focused on epidemiological data [20, 21], nurses’ adherence to protocols [22, 23], nurses’ knowledge of CAUTI-related preventive practices [24–26], and the evaluation of specific interventions [27–30]. While these studies provide valuable information on the scope of the problem and levels of compliance, they offer limited insight into the contextual and experiential factors that shape nursing practice. Specifically, there is a notable gap regarding how nurses perceive the barriers and facilitators to CAUTI prevention within their daily work environment. Furthermore, the potential influence of supervisory practices, interprofessional collaboration, and patient engagement on CAUTI outcomes remains underexplored in the national context.

Building on this perspective, the present study aimed to explore oncology nurses’ perceptions of the barriers and facilitators influencing adherence to urinary catheter-related care standards, as well as to identify their suggestions for improving care in this field. Addressing this knowledge gap is crucial for designing evidence-based interventions that are responsive to local practice contexts. Accordingly, the following research questions were formulated:

- What perceptions do nurses hold regarding current practices for CAUTI prevention?
- What barriers do nurses perceive to the implementation of evidence-based CAUTI prevention practices?
- What strategies do nurses suggest to improve CAUTI prevention practices?

## Materials and methods

This study was part of an action-research project entitled Nursing Practices in the Prevention of Catheter-Associated Urinary Tract Infection [31], initiated in June 2021. The qualitative study was integrated into the action phase of the broader action-research project, as described by Kuhne & Quigley [32]. Data collection was conducted through a focus group. This article adheres to the Consolidated Criteria for Reporting Qualitative Research (COREQ) [33].

### Research team and reflexivity

The focus group was moderated by a male nurse researcher with a PhD in Nursing and extensive experience in infection control-related projects (P.S.-C.). The principal investigator (F.P.-S.), a male MSc holder and nursing professor, observed the session and took field notes. A third team member, a PhD holder and professor of nursing, contributed to the coding and data analysis (L.T.-S.).

The moderator had no previous relationship with the participants. The principal investigator, however, was familiar with the nursing team, as this focus group was part of a broader action research project implemented in their unit. The moderator’s prior experience in infection control provided contextual knowledge, although he had not previously worked specifically in CAUTI.

### Study design

The study adopted a qualitative, exploratory design, using a focus group for data collection and an inductive thematic analysis [34], guided by the three domains of the Nursing Role Effectiveness Model [18]. Coding was conducted inductively without preassigning data to model categories. After initial themes were generated, the Nursing Role Effectiveness Model was used as an organizing framework to interpret and group themes into structure-related and process-related domains.

### Participant selection and recruitment

Purposive sampling was used to select eight nurses from the study setting, in line with international recommendations on focus group size [35]. Selection aimed to ensure diversity of perspectives, including experience in management roles, involvement in infection control activities, professional experience in different hospitals and countries, varied lengths of service in the study setting, and specialization in medical-surgical nursing.

As this study was part of a broader action-research project, the research team requested the support of the unit nurse manager to help identify nurses who had been involved in the project since its beginning. Identified nurses were then contacted directly by the research team, informed about the purpose, time, and location of the focus group, and invited to participate on a voluntary basis. Their involvement was supported during regular working hours, with management assistance in reorganizing care delivery to ensure that patient care was maintained by other members of the team who were not part of the focus group.

All eight nurses agreed to participate (Table 1). The sample included one nurse with previous professional experience abroad, one nurse performing management support functions within the unit, and one nurse acting as the infection control link nurse.

**Table 1:** Demographic and professional characteristics of the study participants

Variables	Study sample (*n* = 8)
**Sex**	
Female	8 (100%)
Male	0 (0%)
**Professional experience** (years)	Mdn = 16.5 years (range: 8–25)
**Professional experience in the study setting** (years)	Mdn 6.5 (1–16)
**Academic degree**	
Bachelor’s	6 (75%)
Master’s	2 (25%)
**Certified specialist nurses (Portuguese Nursing Board)**	
Yes	3 (37.5%)
No	5 (62.5%)

### Setting

This study was conducted in a department of an oncology hospital in Portugal in February 2023. The department admits patients requiring symptom management related to chemotherapy or disease progression, as well as those receiving end-of-life care. In the department, consistent with usual practice in Portugal, urinary catheterization is medically prescribed, where catheter insertion and maintenance primarily performed by nurses; catheter removal is evaluated daily and decided collaboratively by nurses and physicians. Certain catheter-related tasks, such as emptying urine collection bags, may be delegated to healthcare assistants under nurses’ supervision as per local protocols and national regulations [28, 36].

### Data collection and saturation

The semi-structured discussion guide was developed exclusively for this focus group study, in alignment with the study’s objectives, and was validated by three experts in infection prevention and control (F.P.-S., C.B., J.G.). The discussion was structured around the following questions:

1. What is the role of nurses in the prevention of urinary tract-associated urinary tract infection?
2. What are your perceptions of existing practices in the care of people with urinary catheters? What should be improved?
3. What barriers exist to implementing good practices? What facilitators are there to implement good practices?
4. What suggestions would you give to improve practices?

Questions two, three, and four addressed urinary catheter-related care practices and their perceived influence on CAUTI prevention, rather than the occurrence of infection itself.

The focus group was conducted in a private room within the hospital ward, with no non-participants present during the session. Clear “Do Not Disturb” signage was placed outside the room to ensure privacy and prevent interruptions. The focus group was conducted once and lasted 90 min. Given the exploratory nature of the study and the focus on nurses already engaged in the action-research project, the single focus group was considered sufficient to generate rich, practice-based insights aligned with the study objectives. No additional focus groups were conducted due to the scope and phase of the broader action-research project.

The session was audio-recorded using a digital device, and key observational notes were taken by the principal investigator (F.P.-S.). Participants received the full verbatim for review. While they did not provide additional feedback or comments, they confirmed the content reflected their input. The digital recording was permanently deleted after transcript approval.

### Data analysis and reporting

As the focus group was conducted as part of a broader action-research project in which participants were already actively involved, no significant distress or discomfort was anticipated. A debriefing was provided at the end of the session by the moderator, and participants had access to the principal researcher for any follow-up questions or concerns. No financial or material incentives were offered for participation.

The principal researcher (F.P.-S.) transcribed the audio recordings verbatim and anonymized all transcripts before sharing them with the second coder (L.T.-S). Participants had the opportunity to review their own transcript (verbatim) and were invited to report any discrepancies or concerns. Once this member checking process was completed and no changes were requested, the digital audio files were permanently deleted.

Data were analysed using inductive thematic analysis, following the six-phase approach proposed by Braun and Clarke [34]. First, both researchers (F.P.-S. and L.T.-S.) independently familiarised themselves with the data through repeated reading of the transcripts. Second, initial codes were generated inductively, capturing meaningful units related to nurses’ perceptions of urinary catheter care and CAUTI prevention. Third, codes were compared and collated into potential themes through iterative discussion between the researchers.

While coding and theme development were conducted inductively, during the fourth phase themes were organised in relation to the domains of the Nursing Role Effectiveness Model (structure, process, and outcomes) [18]. Within each domain, themes were analytically grouped as facilitators, barriers, or strategies for improvement. The fifth phase involved defining and naming the themes, resulting in a final coding framework (codebook) that integrated inductively derived themes aligned with the model’s domains. This finalized coding framework was applied to the full dataset to ensure consistent categorisation of data segments under the defined themes. Any discrepancies in coding were resolved through consensus between the researchers.

### Ethical considerations

Confidentiality and data protection were strictly maintained throughout the study. Audio recordings were stored on a password-protected computer accessible only to the principal researcher. Prior to the start of the audio recording, participants introduced themselves to the group to support rapport-building. During the session, participants were encouraged to avoid mentioning real names or disclosing identifiable information when discussing clinical cases. At the beginning of the focus group, participants were reminded of the importance of maintaining confidentiality regarding what was discussed, while also being informed that confidentiality among participants could not be absolutely guaranteed. Anonymized transcripts are stored on an encrypted drive for a period of five years and may be made available to third parties upon reasonable request, subject to ethical review and data protection compliance.

## Results

**Fig. 1:**
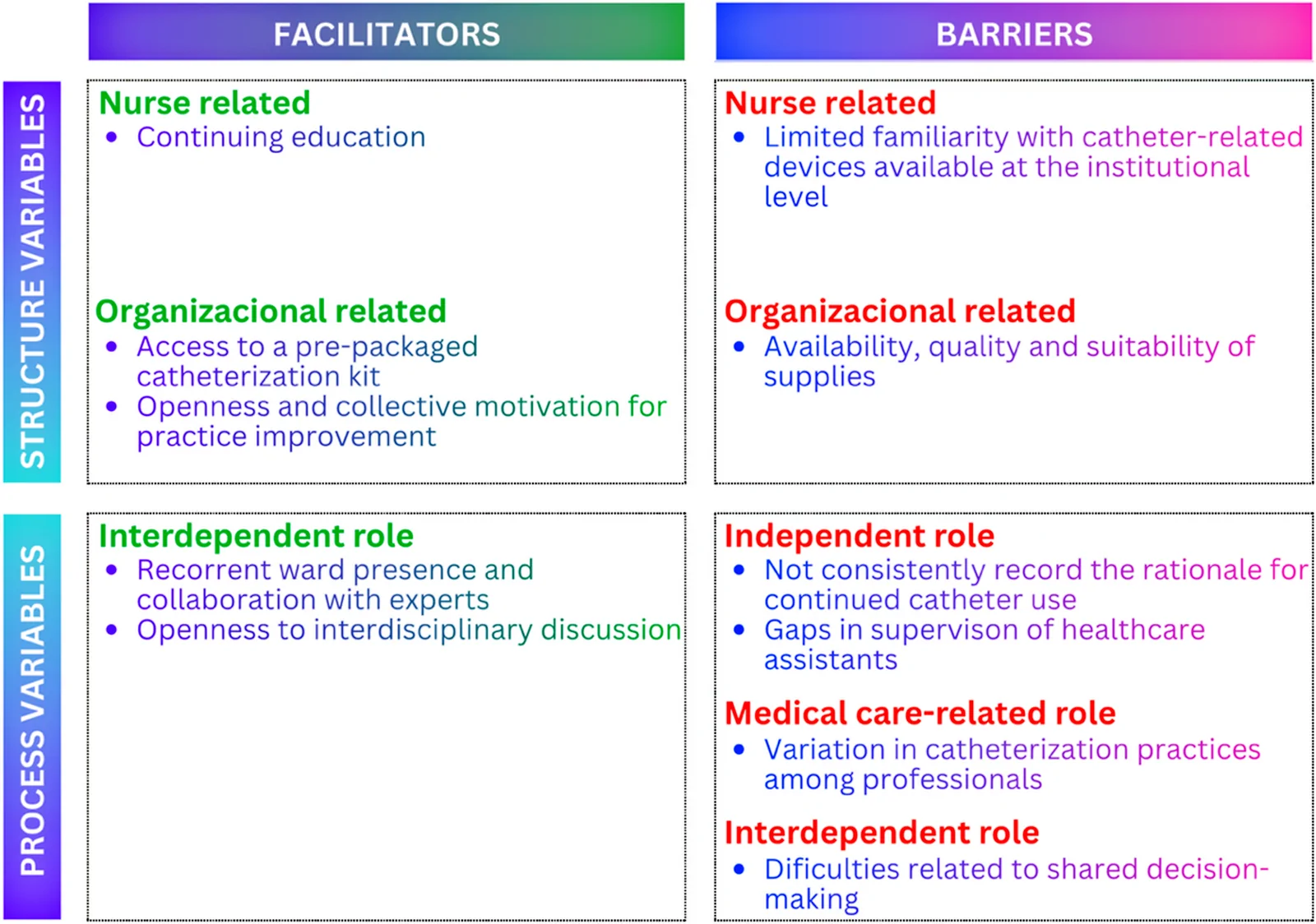
Structure-related and process-related variables identified as facilitators and barriers to CAUTI prevention

The thematic analysis, guided by the Nursing Role Effectiveness Model [18], identified findings across two main analytical domains: structure-related and process-related variables (Fig. 1). These domains represent overarching analytical categories, within which nurses described barriers, facilitators, and strategies for improving CAUTI prevention practices.

### Structure variables

#### Nurse-related variables

Nurses consistently identified continuing education as a key factor supporting improvements in urinary catheter care. Continuing education was described as encompassing both formal academic training (e.g., postgraduate education and specialty courses) and practice-based education, including in-service training sessions and external courses supported by the institution. Participants emphasized that exposure to updated evidence led them to question established routines and abandon outdated practices in daily care:

People who come from other settings and have invested in training also help these practices to take root. (Participant 5, MSN, 16 years of professional experience)

Half of our healthcare assistant team is studying, because their career is also being restructured… there are people who used to only do training at work, and now they’ve sought out courses… those bad practices start to lose ground because the evidence was in plain sight. Knowledge doesn’t come only from experience… it also comes from other sources.(Participant 5, MSN, 16 years of professional experience)

The unit allows us to attend external training… most people have taken specialty courses, post-grads, or other training… there have also been many in-service training sessions. (Participant 7, BSN, 9 years of professional experience)

Focusing on existing barriers, nurses’ limited familiarity with catheter-related devices available at the institutional level was noted as problematic. Participants reported that some materials, such as catheter fasteners, were previously available at the institutional level but were not routinely used in the unit, partly due to limited awareness and insufficient training on their purpose and correct use. This unfamiliarity contributed to variability in practice and delayed adoption of recommended preventive measures.

About the catheter fasteners, I had never seen one before – it was here that I saw a catheter fastener for the first time. (Participant 7, BSN, 9 years of professional experience)

Now we have fasteners… actually, the institution had them, but we didn’t use them in our unit. (Participant 2, BSN, 22 years of professional experience)

#### Organizational-related variables

At the organizational level, nurses described barriers related to quality of care. Participants described institutional issues related to the availability, quality and suitability of supplies as inconsistent. Inadequate or poorly designed catheter-related materials, such as Pen Rose (a well-known brand of external urinary collection device in Portugal) or fixation devices, compromised the effectiveness of catheter care:

With respect to catheter fasteners, these were not available in the unit, and now we do, and all the colleagues use it. (Participant 5, MSN, 16 years of professional experience)

Pen Roses… sometimes having different sizes makes all the difference… the very large ones come off accidentally, and the very small ones can’t even be placed. (Participant 8, BSN, 8 years of professional experience)

We don’t like the available material much, it’s low quality… it comes off easily. We’ve tried using the PenRose on several patients… the good part is we used up the stock of those horrible PenRoses. (Participant 5, MSN, 16 years of professional experience)

We have only one size here… and it’s stiff, very stiff, and doesn’t have a good fixation system – in fact, it’s awful. (Participant 5, MSN, 16 years of professional experience)

As facilitators, having access to a pre-packaged catheterization kit was viewed as a positive tool that enhanced care quality. Participants highlighted the convenience and completeness of the kit, as well as the need to increase its availability within the unit to support consistent aseptic practice.

Everything in the kit we have separately. Of course, it’s much more convenient to grab the kit because you won’t miss any materials. And again, in terms of infection control, it’s better. (Participant 5, MSN, 16 years of professional experience)

When I arrived and saw the kit, I said, ‘Look how wonderful!’ We have everything we need here to maintain an aseptic technique, but under much better conditions. (Participant 2, BSN, 22 years of professional experience)

In the context of catheterization, I actually think that practices here are better… Because we have a kit. (Participant 7, BSN, 9 years of professional experience)

Maybe we should have more [pre-packaged catheterization kits] than what we currently have. (Participant 3, MSN, 25 years of professional experience)

Likewise, nurses reported openness and collective motivation toward practice improvement related to urinary catheter care and CAUTI prevention. A culture of shared engagement, learning, and perceived usefulness of individual contributions was described as enabling the adoption and consolidation of improved practices within the team.

It’s a lack of awareness of that good practice! And I think that once we know it’s a good practice, I think it easily takes root in the team because everyone is open to it… overall, our team gets involved and we’re all interested in learning more and wanting to do better. (Participant 4, BSN, 19 years of professional experience)

It’s about motivation. It’s about involving the largest number of people who feel useful with what they’re doing and showing results… The healthcare assistants feel involved because they are important parts of this process. (Participant 2, BSN, 22 years of professional experience)

With numbers… the infection rate decreased… and they can also see that their work has results. And that’s motivating.” (Participant 2, BSN, 22 years of professional experience)

We’re a team with a good working environment and we have a good relationship with our nurse manager, which facilitates unity towards a goal… we align easily toward a purpose… we’re open to it and function well… there’s unity in achieving goals. (Participant 2, BSN, 22 years of professional experience)

Our team is young and has a critical mindset, and I think people invest in their training… they’re focused on improving practices. (Participant 7, BSN, 9 years of professional experience)

As suggestions for improving CAUTI prevention practices, participants expressed the need for a standardized nursing protocol for urinary catheter care. They believed this would improve consistency and adherence to evidence-based practices.

Create a procedure… let’s create a nursing procedure to standardize this practice so that everyone does it in line with a procedure based on the latest evidence. (Participant 5, MSN, 16 years of professional experience)

So maybe it’s important to standardize this. Cleaning of the urinary meatus is also something I think is starting to become more standardized. (Participant 5, MSN, 16 years of professional experience)

Everyone involved in catheterization [should be involved in creating a protocol]. One person from each category: nurses, doctors, and healthcare assistants. (Participant 3, MSN, 25 years of professional experience)

The catheter you have to keep sterile, and then you place it outside the primary wrapping… maybe that’s another aspect we can consider in the protocol. (Participant 5, MSN, 16 years of professional experience)

Define what the appropriate place is for laying out materials for the procedure. (Participant 2, BSN, 22 years of professional experience)

Of course, we need a workbench… a trolley with a work tray would be easier. (Participant 3, MSN, 25 years of professional experience)

Creating this protocol, and even involving doctors who are willing to work with us, and involving the healthcare assistants.” (Participant 3, MSN, 25 years of professional experience)

Nurses also suggested integrating prompts and alerts into the nursing information system to support and reinforce documentation practices. They emphasized that, although clinical practices had improved, these improvements were not always consistently reflected in nursing records, limiting their visibility and continuity of care:

Maybe we should do it to further improve the numbers and reduce urinary infections. (Participant 8, BSN, 8 years of professional experience)

If it didn’t let you proceed without entering a justification, that would help too. (Participant 4, BSN, 19 years of professional experience)

Generate an alert for the following days and for the other colleagues dealing with the patient. (Participant 3, MSN, 25 years of professional experience)

If there’s no record, it’s like it didn’t happen, that’s a failure, but what’s true is that in daily practice… we talk more… all our practice improved… but we don’t record it… even though it’s visible. (Participant 3, MSN, 25 years of professional experience)

### Process-related variables

#### Independent role

Nurses described aspects of their independent role in urinary catheter management, particularly activities related to clinical assessment, decision-making, and implementation of care measures for which nurses are professionally accountable. Participants acknowledged that a key barrier to this independent role was the inconsistent documentation of the rationale underpinning decisions regarding continued catheter use.

There’s something we still don’t do, which is to record daily, in the patient’s nursing record, the reason for continuing catheterization. Even though it’s something we discuss orally, there’s no written record. (Participant 5, MSN, 16 years of professional experience)

With respect to [nursing] records… I think we don’t mention the reason for catheterization, and we also don’t record daily why it’s maintained. (Participant 8, BSN, 8 years of professional experience)

We are aware that we should do it, but we’re not… there’s no process audit… institutionally, it’s not yet defined as a mandatory record. (Participant 5, MSN, 16 years of professional experience)

Moreover, nurses identified gaps in the supervision of healthcare assistants during delegated catheter maintenance tasks, which they perceived as a barrier to the effective enactment of their independent role in ensuring adherence to hygiene and infection prevention protocols.

There’s a protocol that the healthcare assistants have, which they don’t always follow, that says how they should perform the technique. (Participant 6, BSN, 17 years of professional experience)

They [healthcare assistants] might not do it… because they don’t know, because they’re unaware… they don’t do it because they don’t know there’s another way to do it. (Participant 3, MSN, 25 years of professional experience)

What often fails? It’s the part where they change gloves, wear an apron between patients, clean the end with gauze and something – which is sometimes used just for a drop or two on the floor. (Participant 6, BSN, 17 years of professional experience)

Regarding emptying [the drainage bag by healthcare assistants], I think we should be stricter in monitoring. We should monitor more closely. (Participant 7, BSN, 9 years of professional experience)

They [healthcare assistants] don’t use the equipment they should be using. (Participant 6, BSN, 17 years of professional experience)

They [healthcare assistants] wear gloves, but the problem is that they usually don’t change them between patients. (Participant 6, BSN, 17 years of professional experience)

#### Medical care-related role

Participants identified barriers to their medical care-related role in the field of urinary catheterization. Within this regulated practice context, participants described variation in urinary catheterization care among nurses, referring both to differences in aseptic technique and procedural conduct, and to the inconsistent use of available equipment, such as pre-packaged catheterization kits. These inconsistencies were attributed to habit, lack of standardized protocols, and differing perceptions of appropriate technique.

The procedure is still a bit… it’s still a bit scattered among professionals. Some colleagues use the sterilized kit, and some don’t. (Participant 5, MSN, 16 years of professional experience)

It’s a matter of habit… Because if needed, they’ll even go get the individual pieces, gloves, and bring a drape, but there’s a kit there with everything. (Participant 5, MSN, 16 years of professional experience)

How often do patients have a collection bag with a tap and the bag is changed… we saw that it was replaced because the healthcare assistant didn’t want to handle the urine when emptying into a measuring container, so the assistant simply arrived and changed the bag… our internal protocol is that the assistant only empties, never replaces a bag. (Participant 5, MSN, 16 years of professional experience)

First, we’ve already mentioned here, and it’s the main point… the lack of a protocol. So, the lack of a guide to standardize practice. (Participant 3, MSN, 25 years of professional experience)

Some people use the meal tray, assuming it’s been properly sanitized… many colleagues place materials on the patient’s bed… the patient moves because it’s not exactly a painless procedure… and the material gets contaminated. (Participant 5, MSN, 16 years of professional experience)

#### Interdependent role

As barriers, nurses reported difficulties related to shared decision-making, particularly in interactions with physicians regarding catheter necessity:

Regarding the relationship with the medical team, it’s something I consider negative… this affects the service a lot. Since urinary catheterization involves several interdependent interventions, this fragmentation between teams is harmful. (Participant 7, BSN, 9 years of professional experience)

A patient had a multidrug-resistant infection… there was absolutely no need for catheterization, and the doctor still prescribed it. Even after the colleague responsible for the patient discussed the lack of necessity with the doctor, he disagreed without any justification and didn’t change the prescription. (Participant 5, MSN, 16 years of professional experience)

I think the relationship with doctors could be much better, especially communication-wise… Communication to improve care quality is the most important… they don’t even tell us about aspects that are very important, with implications for the patients, that depend on us. (Participant 2, BSN, 22 years of professional experience)

Nonetheless, nurses identified several potential facilitators to care quality in this field. Recurrent ward presence and collaboration with experts (e.g., researchers, infection control specialists) was reported to positively influence practice change and reflection, while also increasing awareness of improved materials and procedures.

Since the [researcher’s name] started this study and raised awareness among everyone, we started thinking more about the topic and really raising awareness for good practices. Our actions changed, and patient care improved significantly, even just from the questions the [researcher’s name] raised. (Participant 3, MSN, 25 years of professional experience)

That was something the [researcher’s name] mentioned during the training I attended – about keeping the system closed. (Participant 7, BSN, 9 years of professional experience)

We always need help from those who know, who make us aware. Because something only becomes a problem when I realize I’m not doing it right. (Participant 4, BSN, 19 years of professional experience)

Before this study, we would just catheterize and that’s it! Without reflecting so much on these issues. (Participant 4, BSN, 19 years of professional experience)

This collaborative presence was described as a trigger for identifying and considering alternative materials aligned with infection prevention principles:

There are other materials that we became aware of and are trying to bring to the unit, namely the Pen Rose.” (Participant 5, MSN, 16 years of professional experience)

There are other materials we’ve been alerted to that would be beneficial for infection control, namely: catheters with a sampling port for urine collection, so we don’t have to pierce the catheter, which is an entry point for infection. (Participant 5, MSN, 16 years of professional experience)

Another facilitator concerned the ward team’s openness to interdisciplinary discussion. Participants described greater comfort engaging in clinical dialogue with physicians. They saw increased knowledge as empowering them to advocate for patient care improvements:

The fact that we now question doctors more openly, and that our increased knowledge gives us confidence, allows us to discuss with the medical team the need for urinary catheter use, including decisions about insertion, removal, and related procedures. (Participant 3, MSN, 25 years of professional experience)

Nonetheless, as suggestions for care improvement, the participants expressed interest in more structured opportunities for expert-led case management reflection and discussion in this field:

If we promote this kind of reflection [expert-led discussion and case-based reflection], like what the [researcher’s name] came here to do. (Participant 4, BSN, 19 years of professional experience)

“I think having these moments of reflection, for this or any other reason, is important for the team because it always gets us thinking more about things.” (Participant 8, BSN, 8 years of professional experience).

## Discussion

This study explored nurses’ perceptions of barriers, facilitators, and improvement strategies related to urinary catheter care and CAUTI prevention. Overall, the findings highlight how nurses’ professional roles, responsibilities, and interactions within the care team shape both opportunities and constraints for delivering evidence-based catheter care. Our findings support that challenges in supervision, organizational conditions, learning processes, and the articulation of nurse-sensitive outcomes shape how CAUTI prevention is enacted in practice.

One prominent example of how nurses’ roles and responsibilities shape care quality emerged in relation to the supervision of healthcare assistants during delegated catheter-related tasks. A key finding was the tension between nurses’ identification of barriers located in healthcare assistants’ practices and the relatively limited explicit attention given to nurses’ own supervisory role in relation to these delegated activities. These findings extend previous research by showing that supervisory responsibilities are often embedded in everyday nursing practice rather than explicitly recognized as a distinct area of reflection [37].

Nurses frequently expressed concerns that healthcare assistants did not uniformly follow evidence-based infection control protocols (e.g., during urine bag management). However, despite Portuguese nursing regulations emphasizing that delegation requires adequate preparation, supervision, and accountability [38], nurses did not explicitly acknowledge their role in ensuring the quality of these delegated practices. This suggests a potential relational blind spot, wherein suboptimal care delivered by assistants is viewed as external to the nursing role, rather than as an outcome influenced by the effectiveness of supervision and leadership. Prior studies have shown that nurses’ knowledge and practices regarding CAUTI prevention may be suboptimal [39], and that clear role delineation and interprofessional accountability are essential for prevention [40]. Addressing this gap through targeted education on leadership, delegation, and supervisory skills may enhance consistency and accountability in CAUTI-related care [41, 42].

Second, the results indicate a strong organizational and nurse-centric orientation in how prevention was conceptualized. Nurses identified structural barriers (e.g., inconsistent material availability, suboptimal electronic health record prompts) and process barriers related to interprofessional communication. The lack of material resources and low interprofessional communication was identified in a previous study in the same setting [43]. In contrast, participants did not identify patient or family involvement as either a facilitator or a barrier to care. While participants valued informing patients of the catheter indication and obtaining consent, there was little mention of teaching about self-care practices, early symptom recognition, or promoting patient participation in catheter management and infection prevention. This omission suggests a relatively narrow conception of CAUTI prevention as a nurse-driven procedural task, rather than as an opportunity for shared care with patients and families [44]. Given that patient education and engagement are known to contribute to better infection prevention outcomes [45, 46], expanding nursing practice to more systematically incorporate patient-level interventions could further strengthen prevention efforts.

Our findings suggest that situated learning and reflective practice were deemed powerful facilitators of change. Participants described how their awareness of CAUTI risks and preventive strategies had improved substantially through the action research project’s reflective discussions. These moments of dialogue and collaborative reflection seemed to prompt deeper understanding and greater ownership of best practices than traditional top-down training alone. This finding aligns with prior research showing that interactive, team-based learning supports sustainable behavior change in healthcare [47, 48]. Embedding regular opportunities for reflective practice within clinical teams may therefore be a valuable strategy to maintain and further enhance CAUTI prevention practices [49, 50].

Finally, the data suggest that nurses in this setting may hold a narrow view of nurse-sensitive outcomes, focusing largely on procedural correctness and documentation rather than on broader patient-centered outcomes. While this is consistent with the organizational emphasis on adherence to institutional protocols described in previous studies [51–53], it risks underemphasizing the importance of patient empowerment, health literacy, and self-management capacity — all of which can influence infection risks and outcomes after discharge.

Developing a more expansive understanding of nurse-sensitive outcomes [54], one that explicitly links nursing interventions to patient experiences and longer-term results, may help nurses to see patient education and engagement as integral components of CAUTI prevention [55]. Importantly, the study also highlighted the presence of a team culture characterized by openness to learning and collaborative improvement. This cultural strength represents an important enabler for future interventions and should be leveraged to foster sustained enhancements in CAUTI prevention practices.

### Limitations

This study has some limitations. First, the findings are based on a single focus group conducted within one oncology hospital department in Portugal. While participants represented diverse professional backgrounds and experiences, the results do not necessarily reflect the views of nurses in other clinical contexts or specialties. Second, as the focus group was conducted during the action phase of an action-research project in which participants were already engaged, prior exposure to project-related discussions likely influenced the content and tone of participants’ responses. Third, while efforts were made to promote open discussion, social desirability bias may have led some participants to underreport personal or team-related barriers. Despite these limitations, the study offers valuable insights into the lived experiences and perceptions of Portuguese nurses actively engaged in CAUTI prevention, which has not been addressed in previous research at a national level. Further studies in different Portuguese clinical contexts would help explore how these findings may reflect broader patterns of nursing practice in CAUTI prevention.

## Conclusions

This study explored oncology nurses’ perceptions of barriers and facilitators to urinary catheter-related care, with the aim of informing improvements in CAUTI prevention. Our findings suggest that while nurses recognize key structural and process-related barriers, they also demonstrate a strong commitment to practice improvement. Gaps remain in the supervision of delegated tasks and in the engagement of patients and families as active partners in care. Given participants’ repeated emphasis on the absence of clear guidance, the development and implementation of standardized, evidence-based protocols for urinary catheter care emerges as a key recommendation to support consistent practice. Promoting reflective practice, broadening how nurse-sensitive outcomes are conceptualized, and strengthening supervisory and educational strategies may support more comprehensive and sustainable approaches to CAUTI prevention in clinical nursing practice.

## Data Availability

The datasets analyzed during the current study are not publicly available due to participant privacy concerns but are available from the corresponding author upon reasonable request.

## Abbreviations

CAUTI: Catheter-associated urinary tract infection
BSN: Bachelor of Science in Nursing
MSN: Master of Science in Nursing
